# The Family Table

**Published:** 1883-05

**Authors:** 


### THE FAMILY TABLE.

THE influence and the effect upon character of all that pertains to the family table has been remarked upon so often that one would think there could be nothing more to say in regard to it, but there is one point to which mothers might profitably give thought, and that is the culture their children here receive of generosity or of stinginess. If you doubt whether this may be done, sit down and call to mind what you know of the inner life of some households, and see if one person of your acquaintance of generous and liberal ideas ever came out of a home which was, as Mr. Emerson says, "built to the end of prudence" alone; where the amount of meat provided was never sufficient to quite satisfy; where the number of potatoes exactly corresponded to the number of the family; where the biscuits were so small that two would not take the place of one goodsized one and yet no one would dare ask for three; where the cookies are thin as wafers, and only serve to remind the children that there are such things as cookies in the world; where to go on a visit which may legitimately end in "staying to tea, if they ask you," is a subject of the utmost importance to the greedy-eyed children; where the cost of each article of food is commented upon, and the idea conveyed that the chief end of man is to fill the stomach at the least possible outlay of capital. For my part I believe that the effect upon the young of such a table is to be traced in after life, and with a distinctness that is startling. It may be said that the children inherit the tendencies of the parents, which is of course true; but the point here made is that tendencies may be cultivated or repressed. "The world will never know," said a gentleman the other day, "what I have thought and suffered on account of cherry pie. I was never allowed more than one very small piece, and have gone through life 'feeling,' as Tennyson says, 'where I cannot prove' that there is something wrong in my having all I want of it."

DRINKING WATER.—The inhabitants of European countries seldom drink water clear; they usually add a little sugar to it, not only to render it more palatable, but more wholesome. There is a popular idea that sugar nutralizes any impurities that may exist in the water, thus depriving it of the power to contaminate the system and induce disease.

